# Comorbidity of Type 1 Diabetes and ADHD: A Longitudinal Cohort Study in Males and Females From the Norwegian Childhood Diabetes Registry

**DOI:** 10.1155/pedi/9574797

**Published:** 2025-11-10

**Authors:** Kristin Andersen Bakke, Cathrine Brunborg, Egil Midtlyng, Sissel Berge Helverschou, Torild Skrivarhaug

**Affiliations:** ^1^Division of Paediatric and Adolescent Medicine, Oslo University Hospital, Oslo, Norway; ^2^Institute of Clinical Medicine, Faculty of Medicine, University of Oslo, Oslo, Norway; ^3^Oslo Diabetes Research Centre, Oslo, Norway; ^4^Oslo Centre for Biostatistics and Epidemiology, Oslo University Hospital, Oslo, Norway; ^5^The Norwegian Childhood Diabetes Registry, Oslo University Hospital, Oslo, Norway

## Abstract

**Aims:**

This study of interactions between attention-deficit hyperactivity disorder (ADHD) and type 1 diabetes has two aims. First, compare the prevalence of prescribed ADHD medications in individuals with type 1 diabetes to the general paediatric population in Norway. Second, study trajectories in mean glycated haemoglobin (HbA1c) in individuals with comorbid ADHD in comparison to individuals with type 1 diabetes only.

**Methods:**

Part 1 uses data from the Norwegian Prescription Database and register linkage between the Norwegian Childhood Diabetes Registry (NCDR) and the Norwegian Prescribed Drug Registry to obtain yearly prevalence in the general population and NCDR between 2005 and 2019. Part 2 uses data from annual controls in NCDR between 2005 and 2022. Linear mixed-effects models adjusted for age and diabetes duration were used to compare mean HbA1c in 365 individuals (66% males) with comorbid ADHD to 5,888 individuals (54% males) with type 1 diabetes only.

**Results:**

Part 1: Yearly prevalence (2009–2019) ranged from 0.02 to 0.52 percentage points higher in NCDR than in the general population. The difference was significant only for 2017. Part 2: Mean national HbA1c decreased from 2012 to 2022 but was higher in individuals with comorbid ADHD. This difference was significant from 2016 (67.6 mmol/mol [8.3%] vs. 64.5 mmol/mol [8.1%]) to 2022 (57.5 mmol/mol [7.4%] vs. 54.7 mmol/mol [7.2%]). When grouped by sex, these differences continued to be significant in males (except in 2018), but not in females. Individuals with ADHD were more likely to have experienced at least one episode of diabetic ketoacidosis (odds ratio [OR] = 1.39, 95% confidence interval [CI] [1.04, 1.86]; males: OR = 1.65, 95% CI [1.15, 2.36]; females: OR = 1.10, 95% CI [0.658, 1.83]).

**Conclusion:**

Mean national HbA1c decreased in Norway during the last decade, but continued to be higher in individuals with comorbid ADHD than in individuals with type 1 diabetes only.

## 1. Introduction

Type 1 diabetes and comorbid attention-deficit hyperactivity disorder (ADHD) is a challenging combination both for individual patients as well as for health care professionals [1, 2]. Some authors have argued that specially adapted care might be needed [3]. Individuals with a dual diagnosis of type 1 diabetes and ADHD have been shown to have poor metabolic control and increased risk of diabetic ketoacidosis and severe hypoglycaemia compared to individuals with type 1 diabetes without comorbid ADHD [1, 2]. In addition, studies have reported a higher prevalence of ADHD in individuals with type 1 diabetes compared to the general population [2, 4].

Type 1 diabetes is a severe lifelong autoimmune disease characterised by loss of endogenous insulin production. Treatment of type 1 diabetes is challenging, and individuals need to master attention-demanding tasks such as frequent blood glucose monitoring, carbohydrate counting, considering insulin dose and insulin administration in association with meals, as well as adjustments for physical activity [1, 5]. ADHD is a neurodevelopmental disorder characterised by an ongoing pattern of inattention and/or hyperactivity and impulsivity that impairs functions in two or more settings. Several symptoms should be present before 12 years of age [6, 7]. More males than females are diagnosed with ADHD [8]. ADHD may interfere with a person's ability to manage type 1 diabetes, which again mediates higher glycated haemoglobin (HbA1c) in individuals with dual diagnosis [1].

Disentangling the association between type 1 diabetes, ADHD and poor metabolic control is difficult, and it can be challenging to recognise ADHD in individuals with type 1 diabetes. Executive functions describe a set of skills that are needed for independent, purposeful, goal-directed behaviours, which are essential for successful adherence to type 1 diabetes treatment [9–11]. It has been well documented that individuals with ADHD have deficits in executive functions [12]. Impaired neuropsychological functions in ADHD, such as a deficit in executive functions, may affect the ability to master type 1 diabetes treatment [1]. However, it may also be the other way around; impaired glucose regulation may, by itself, impair cognitive abilities and, in particular, executive functions. Several studies, including one longitudinal study, have shown an association between executive functions, treatment adherence and glycaemic control [10, 11, 13]. Moreover, impaired glycaemic control, especially chronic hyperglycaemia, has been reported to be injurious to the developing brain [13]. Some authors have discussed the possibility of a negative feedback loop when impaired executive functions lead to suboptimal treatment adherence, which again could cause dysglycaemic brain insults and in turn lead to further impaired executive functions [13]. A Swedish study reported that poor glycaemic control was associated with an increased risk of subsequent diagnosis of neurodevelopmental disorders, particularly ADHD [14].

HbA1c reflects average plasma glucose in the preceding 8–12 weeks, and is regarded as the most important quality indicator in type 1 diabetes [5, 15]. Optimising glycaemic control is essential to prevent micro- and macro-vascular diseases, and there is definitive evidence of the association between HbA1c and the development of diabetes late complications [16, 17].

In Norway, the mean national HbA1c has improved in children during the last 10 years [18], but we do not know if individuals with type 1 diabetes and comorbid ADHD have the same improvements in metabolic outcomes as individuals with type 1 diabetes only [18]. Also, we do not know whether individuals with type 1 diabetes are diagnosed and treated with ADHD medications at the same rate as the general paediatric population. This is the first national study assessing ADHD in children with type 1 diabetes in Norway.

The main aims of this study were: (1) to compare the yearly prevalence of prescribed ADHD medications in individuals with type 1 diabetes to the yearly prevalence of prescribed ADHD medications in the general paediatric population in Norway. (2) To investigate if there were different trajectories in the national mean HbA1c in those with and without comorbid ADHD.

## 2. Materials and Methods

### 2.1. Study Design and Materials

This population-based, nationwide longitudinal cohort study consists of two parts: Part 1 reports the yearly prevalence of prescribed ADHD medications in the general paediatric population and in individuals with type 1 diabetes. Part 2 compares metabolic control and treatment modalities in individuals with type 1 diabetes with and without comorbid ADHD.

Part 1 uses data from the Norwegian Prescription Database, which is a national open pseudonymous registry provided by the Norwegian Institute of Public Health that contains data about prescribed medications. Data can be split by sex and by 5-year interval age groups (0–4, 5–9, 10–14 years, etc.). Both parts 1 and 2 use data from register linkage between the Norwegian Prescribed Drug Registry and the Norwegian Childhood Diabetes Registry (NCDR). The Norwegian Prescribed Drug Registry is a personally identifiable registry containing data about all dispensed drugs in Norway. NCDR comprises data about all children aged 0–17 years with diabetes in Norway and has a high coverage rate, 99% at diagnosis and 98% at annual follow-ups [19]. In Norway, all children with type 1 diabetes are treated in a paediatric department up to age 17 years. NCDR receives standardised data at diagnosis and later from annual controls from all the hospitals. The Norwegian Institute of Public Health performed the register linkage in this study. Personal identification numbers were replaced by code, and data was de-identified.

### 2.2. Participants

The study cohort comprised individuals in NCDR who were diagnosed with type 1 diabetes between January 1, 2004, and December 31, 2021, and who had registered at least one annual control in NCDR between January 1, 2005, and December 31, 2022. We found 255 individuals (176 males, 69%) who had received at least one reimbursable prescription of ADHD medication (dexamphetamine, methylphenidate, atomoxetine, lisdexamfetamine or guanfacine) between January 1, 2005, and December 31, 2019.

Part 1 comprises data about prescribed ADHD medication between 2005 and 2019 in the general paediatric population and in NCDR. We used data from the Norwegian Prescription Database to find the yearly prevalence of ADHD medication in the general paediatric population, 5–19 years (Table 1). Data obtained by register linkage were used to find the yearly prevalence of prescribed ADHD medication in NCDR. The Norwegian Prescribed Drug Registry comprised data on age, sex, type of prescribed ADHD medication and date of when medication was dispensed. Table 1 shows the number of individuals in our cohort, ≥ 5 years of age, with annual control in NCDR each year between 2005 and 2019 and the number of individuals who had been prescribed ADHD medication the same year.

**Figure 1:**
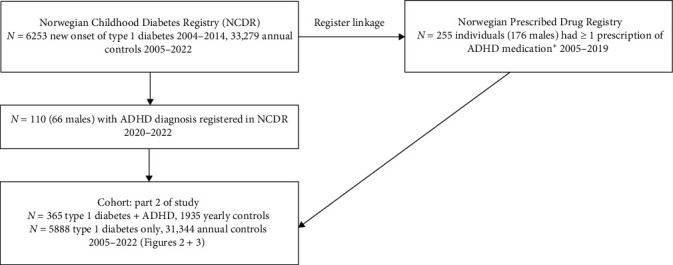
Flow chart showing recruitment procedure of the cohort. *⁣*^*∗*^ADHD medication with the following ATC-codes: N06B A02, dexamphetamine; N06B A04, methylphenidate; N06B A09, atomoxetine; N06B A12, lisdexamfetamine; C02A C02, guanfacine.

Part 2 uses data from the annual controls in NCDR between 2005 and 2022. All 255 individuals who had been prescribed ADHD medication were considered to have ADHD. It has been possible since January 1, 2016, to record in NCDR when an individual has received an ADHD diagnosis by the specialist health services. An additional 110 individuals (66 males, 60%) with ADHD were identified using data from NCDR between 2020 and 2022. Since ADHD is a neurodevelopmental disorder starting before 12 years of age [7], we considered individuals to have comorbid ADHD on all annual controls, including controls prior to the first ADHD prescription or prior to ADHD being first registered in NCDR. The use of ADHD as a lifetime diagnosis has been used by other authors investigating ADHD in type 1 diabetes [20]. The total cohort comprised 6253 individuals with type 1 diabetes. There were 365 (5.8%) individuals with type 1 diabetes and comorbid ADHD who attended 1935 yearly controls and 5,888 individuals with type 1 diabetes without comorbid ADHD who attended 31,344 annual controls.  Figure 1 illustrates the recruitment procedure. All patients had at least 6 months of diabetes duration and 1 yearly control before the study period ended on December 31, 2022.

### 2.3. Ascertainment of ADHD

In Norway, diagnostic assessments of ADHD are done only within the specialist health services. The Norwegian clinical guidelines state that the criteria of the current version of the Diagnostic and Statistical Manual of Mental Disorders (DSM-5 and DSM-IV before 2013) should be used. Prescription of ADHD medication can only be initiated by doctors working in the specialist health service, but further medical treatment of ADHD can be continued by primary care physicians. From 2009, doctors in Norway used diagnostic codes on the prescription to ascertain that the patient had ADHD. Prior to 2009, doctors coded that the patient had a chronic condition for which prescribed medication was reimbursable.

### 2.4. Variables of Interest

The annual controls in NCDR are standardised examinations which include anamnestic, clinical, and laboratory variables. We used the following variables from 2005 to 2022: HbA1c, age, sex, diabetes duration, diabetic ketoacidosis and severe hypoglycaemia with loss of consciousness with or without convulsions. All HbA1c samples in the annual controls were analysed at a single national Diabetes Control and Complications Trial (DCCT)-standardised laboratory (Aker, Oslo University Hospital). NCDR also includes data on treatment modalities, insulin pumps or insulin pens, and, from 2016, we also have data on active carbohydrate counting and the use of continuous glucose monitoring (CGM).  Table 2 presents some characteristics of individuals at the time of inclusion in part 2 of the study.

### 2.5. Statistical Analyses

For each year between 2005 and 2019, we calculated the prevalence of ADHD prescriptions in the total group of individuals aged 5–19 years, 5-year age groups, males, and females, in the general population and in NCDR. Yearly and the average prevalence of ADHD prescriptions between 2005 and 2019 in NCDR were compared to the prevalence in the general paediatric population. We used the *Z*-score calculator for two population proportions (socscistatistics.com), two-tailed hypotheses to calculate *p*-values.

Descriptive statistics are presented as frequencies with percentages or means with standard deviation (SD) as appropriate. Differences in continuous variables between groups were tested with the Student's *t*-test for normally distributed data. Chi-square test for contingency tables was used to detect differences in categorical variables.

At annual controls in NCDR, few individuals were recorded as having experienced diabetic ketoacidosis or severe hypoglycaemia in the last year. These data were therefore dichotomised into having or not having ever experienced diabetic ketoacidosis or severe hypoglycaemia between 2005 and 2022. Data concerning treatment were dichotomised into used/not used insulin pump, active carbohydrate counting, and CGM. Logistic regression analyses were performed to investigate the association between type 1 diabetes with and without comorbid ADHD and diabetic ketoacidosis or severe hypoglycaemia in the last year (as outcomes), and to adjust for sex and number of years of follow-up in the study. Subsequently, the analyses were repeated within strata of males and females. In addition, we report the *p*-value of the ADHD-by-sex interaction. The results are presented as odds ratios (ORs) with 95% confidence intervals (CIs).

Linear mixed-effects models were used to investigate the relationship between ADHD and HbA1c as the outcome variable to account for repeated measures by patient. Time, group (ADHD), covariates, and time-by-group interaction were fixed effects in all models. All models included random intercept and slope, and an unstructured covariance matrix was used. The models were adjusted for age and diabetes duration. Based on the linear mixed-effects model, we estimated mean values with 95% CIs for the time points for each group. We also estimated mean within-group and between-group differences in change from 2005 to 2022. The analyses were repeated within strata of males and females.

A significance level of 5 % was used. Statistical analyses were performed using the IBM SPSS Statistics version 29.0 (IBM SPSS Inc., Armonk, NY: IBM Corp) and STATA 18.0 (StataCorp LP, College Station, TX).

## 3. Results

### 3.1. Results Part 1

The national yearly prevalence of prescribed ADHD medications increased between 2005 and 2019 (Table 1). For each year starting in 2009, prevalence rates of prescribed ADHD medication were slightly higher in NCDR than in the general population. The prevalence ranged from 0.02 to 0.52 percentage points higher in NCDR than in the general population, but when comparing yearly prevalence, the finding was only significant for 2017 (2.64% vs. 2.02%, *p*=0.0271; Table S1). Average yearly prevalence of prescribed ADHD medications between 2005 and 2019 was slightly higher among individuals with type 1 diabetes than in the general population, but this finding was not significant (2.19% vs. 1.83%, *p*=0.289). More males than females were treated for ADHD in both the NCDR and the general population.

Among individuals with type 1 diabetes, methylphenidate was the most frequently prescribed ADHD medication, and 97% had been prescribed methylphenidate, including 58% who had been prescribed only methylphenidate. There were 21% who had been prescribed atomoxetine, 19% lisdexamfetamine, 2% dexamphetamine and 3% guanfacine. Mean age at first ADHD prescription was lower in males (10.9 years, SD: 3.9) than females (12.3 years, SD: 4.2) *p*=0.004. There were 13 individuals (7 males) who received their first prescription of ADHD medications when they were 18 years or older.

### 3.2. Results Part 2

**Figure 2:**
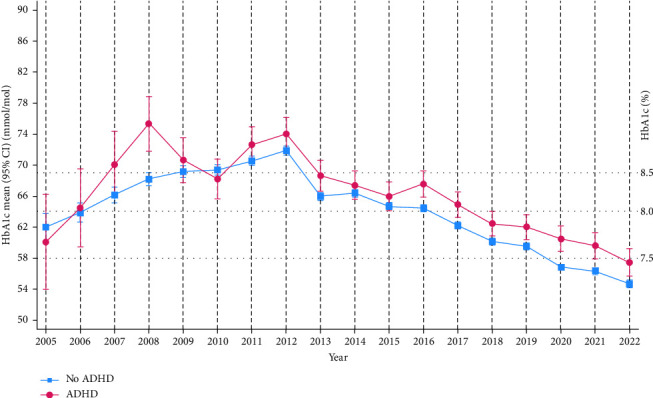
Graph showing mean HbA1c with 95% confidence intervals for time points in individuals with type 1 diabetes with and without comorbid ADHD estimated with a linear mixed effects model adjusted for age and diabetes duration between 2005 and 2022.

**Figure 3:**
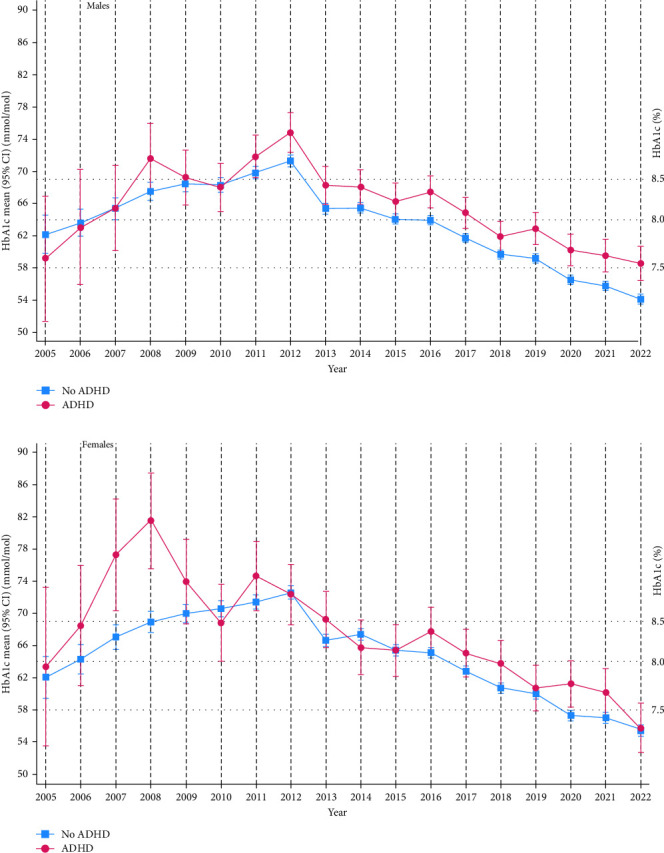
Graphs showing mean HbA1c with 95% confidence intervals for time points in (a) males and (b) females with type 1 diabetes with and without comorbid ADHD estimated with a linear mixed effects model adjusted for age and diabetes duration between 2005 and 2022.

The comparison of metabolic control and treatment parameters in individuals with type 1 diabetes with and without comorbid ADHD showed that HbA1c decreased in both groups from 2012 to 2022 (Figure 2). Over the entire follow-up time (2005–2022), the reduction in HbA1c was −2.62, 95% CI (−8.97, 3.72) in the ADHD group and −7.29, 95% CI (−9.15, −5.44) in those without comorbid ADHD (mean between-group difference 4.67, 95% CI [−1.86, 11.2], *p*=0.161). Mean national HbA1c was higher in individuals with dual diagnosis than in individuals with type 1 diabetes only, and this difference was significant from 2016 (67.6 mmol/mol, 95% CI [65.9, 69.3], vs. 64.5 mmol/mol, 95% CI [64.0, 64.9], to 2022 (57.5 mmol/mol, 95% CI [55.7, 59.2] vs. 54.7 mmol/mol, 95% CI [54.2, 55.2]). When grouped by sex, these differences continued to be significant in males (except for 2018), but not in females (Figure 3). The estimated overall mean difference with 95% CI in HbA1c between those with and without ADHD showed that those with ADHD, on average, over the entire follow-up period had higher HbA1c levels than those without ADHD (mean difference = 2.46, 95% CI [1.33, 3.58]). Stratified analysis in males showed a mean difference = 2.86, 95% CI (1.53, 4.26), and in females, a mean difference = 2.13, 95% CI (0.17, 4.09). We found no interaction effect of sex (*p*=0.331).

A total of 763 individuals (382 males) had experienced one or more episodes of diabetic ketoacidosis during follow-up for their diabetes, but the majority (522 individuals) had experienced only one episode. The risk of having experienced diabetic ketoacidosis was higher in individuals with comorbid ADHD than in individuals with type 1 diabetes only (OR = 1.39, 95% CI [1.04, 1.86]). This effect remained significant after adjusting for sex and number of years of follow-up in the study. Stratified analysis with respect to sex showed that males, but not females, with comorbid ADHD had increased odds of having experienced diabetic ketoacidosis (males: OR = 1.65, 95% CI [1.15, 2.36]; females: OR = 1.10, 95% CI [0.658, 1.83]). We found no interaction effect of sex (*p*=0.202).

A total of 881 individuals (467 males) had experienced at least one episode of severe hypoglycaemia, and there was no difference in odds between individuals with comorbid ADHD and type 1 diabetes, neither for the total group nor when stratified by sex (OR = 1.14, 95% CI [0.85,1.52], males: OR = 1.12, 95% CI [0.84,1.72], females: OR = 1.06, 95% CI [0.64,1.75]). We found no interaction effect of sex (*p*=0.702).

From 2016 to 2022, individuals with comorbid ADHD were more likely to use insulin pumps than individuals with type 1 diabetes only (89.7% vs. 84.4%, *p*=0.014). Individuals with and without comorbid ADHD were equally likely to use CGM (84.7%) and carbohydrate counting more than 50% of the time (83.0%).

## 4. Discussion

The study consists of two parts. First, we reported that the prevalence of prescribed ADHD medications between 2009 and 2019 tended to be higher in NCDR than in the general paediatric population. However, yearly prevalence was significantly higher only in 2017. Second, we investigated the trajectories of mean HbA1c and found that mean HbA1c adjusted for age and diabetes duration had declined during the last decade but continued to be slightly higher in individuals with a dual diagnosis of ADHD and type 1 diabetes than in individuals with type 1 diabetes only.

Our finding that the yearly prevalence of prescribed ADHD medication was slightly higher in individuals with type 1 diabetes seems to align with other studies. Different studies, including two systematic reviews and meta-analyses [2, 4], have reported that individuals with type 1 diabetes have an increased chance of being diagnosed with ADHD [14, 21, 22]. One of the reviews stated there was an overall 35% increase in the prevalence of ADHD among patients with type 1 diabetes (OR = 1.35, 95% CI [1.08, 1.73]) [2]. Moreover, numbers vary between studies, and different authors have employed various methodologies, cohorts, and age groups to study ADHD in individuals with type 1 diabetes. Our study can be compared to a German study that used a prescription database and reported a prevalence of ADHD prescriptions of 2.9% in children with diabetes, compared to 2.4% (*p*=0.004) among children without diabetes [23]. However, it could be noted that the German study included children from 0 to 17 years, and the prevalence of prescribed ADHD medication was higher in the non-diabetic group in children who were from 12 to 17 years old.

Diverse hypotheses have been proposed to explain the observation of a higher prevalence of ADHD in type 1 diabetes that is reported in several studies. Some have said that children with type 1 diabetes are more likely to be evaluated and receive an appropriate ADHD diagnosis since they are seen more frequently by health care providers than the general population [23]. Others have discussed the possibility that autoantibodies could be important not only in the development of type 1 diabetes but also in a subgroup with ADHD [4, 24, 25], and some have highlighted the possibility of common genetic factors linked to both type 1 diabetes and ADHD [4, 21]. On the contrary, Butwicka et al. [22] reported no increase in ADHD in siblings of children with type 1 diabetes. Then again, some argue that diabetes-related factors could lead to altered neuropsychological functions and subsequent ADHD diagnosis [14, 21, 22]. This hypothesis seems to correspond with studies that have investigated the effect of type 1 diabetes on the developing brain [13], and suggests that type 1 diabetes is an independent risk factor for ADHD, particularly in individuals with high HbA1c. Moreover, this view challenges the assumption of ADHD being mainly due to genetic factors in the general population [14].

Table 1 shows the increasing prevalence of prescribed ADHD medications during the study period, and this finding is in line with other studies showing that consumption of ADHD medications has increased worldwide [26]. Yearly prevalence of prescribed ADHD medication was higher in males than in females both in the general population and in NCDR. It is well known that females are less likely to be diagnosed with ADHD, and growing evidence suggests that the sex difference at least partly can be explained by underrecognition of ADHD in females, including late or overlooked diagnosis [8]. According to a recent review, females are typically diagnosed with ADHD later in life than males and are also less likely to be prescribed ADHD medications when diagnosed [8]. This seems to fit with our data, as we found that the average age of first ADHD prescription was higher in females than in males.

A diagnosis of ADHD does not warrant the immediate start of ADHD medications since non-pharmacological interventions are first-line treatments, and it can be noted that 13 individuals (7 males) in our study received their first prescription of ADHD medications at the age of 18 years or older. We do not know what kind of ADHD symptoms were evident in these individuals before 12 years of age, but one may wonder whether diabetes related brain changes could have contributed so that symptoms of ADHD became more evident with increasing age and medical treatment, therefore became necessary.

To our knowledge, part 2 of this study is the first to compare longitudinal trajectories in mean HbA1c in individuals with type 1 diabetes with and without comorbid ADHD in a nationwide study. We found that during the last 10 years, the national mean HbA1c has improved in children with type 1 diabetes, both with and without comorbid ADHD, but individuals with dual diagnosis had higher mean HbA1c than individuals with type 1 diabetes only. The size of the differences in national mean HbA1c between individuals with type 1 diabetes with and without comorbid ADHD in our study (3.1 mmol/mol in 2016 and 2.8 mmol/mol in 2022) was slightly smaller than the differences reported by others [1, 2], but our results were adjusted for age and diabetes duration. The differences may still be clinically relevant since the mean HbA1c in both groups was higher than the recommended treatment goal of HbA1c < 53 mmol/mol. Individuals with dual diagnosis had a higher risk of having experienced diabetic ketoacidosis, and together these findings support the notion that individuals with type 1 diabetes and comorbid ADHD struggle to achieve good metabolic control [1, 2, 27, 28].

As illustrated in Figure 3, from 2016, there was a clear and significant difference in trajectories in mean HbA1c between males with and without comorbid ADHD, whereas, as illustrated by the overlapping CIs, the difference in HbA1c in females with and without comorbid ADHD was not clear. Similarly, the OR for having experienced diabetic ketoacidosis in individuals with comorbid ADHD versus individuals with type 1 diabetes only was significant in males but not in females. These non-significant findings in females might be related to the lower number of females (*n* = 123) than males (*n* = 242) with type 1 diabetes and comorbid ADHD in our study, but we do not know whether ADHD was overlooked in females. Moreover, a systematic review states that females have a higher risk of diabetic ketoacidosis than males [29].

Since impairment in executive functions is both a feature of ADHD and associated with increased HbA1c, one may wonder if there is a risk that an underlying ADHD may not be properly recognised in those with type 1 diabetes who struggle to achieve HbA1c within the recommended range. Some authors have suggested screening for ADHD when type 1 diabetes is diagnosed and then performing repeated screening, particularly in those with poor metabolic control [14, 30, 31]. Moreover, different international studies, including a large register study from Norway, have shown that females have higher HbA1c than males [18, 29], and females seem to be more likely to present with the inattentive subtype of ADHD instead of combined or hyperactive-impulsive subtypes [8]. We can only speculate whether the likelihood of having a higher HbA1c and more often presenting with the inattentive subtype of ADHD in females, make clinicians less likely to look for underlying ADHD in females with high HbA1c.

The improvement in HbA1c seen over the last 10 years in Norway has been associated with improvement in diabetes technology and carbohydrate counting [18, 32]. There were no differences in the use of CGM or carbohydrate counting, but individuals with comorbid ADHD were more likely to use pumps. This could suggest that individuals using insulin pens who have HbA1c within the target range were less likely to be encouraged to change their treatment regimen. Our results indicate that individuals with comorbid ADHD may need special support from health care professionals in order to manage their diabetes treatment and fully benefit from the use of advanced diabetes technology [1, 3, 14, 27].

This study has some limitations. Individuals with an ADHD diagnosis who had never received ADHD medication were not identified by register linkage. This number is unknown, but is supposed to be low since most individuals with an ADHD diagnosis are likely to have tried ADHD medication at least once. Moreover, the prevalence of prescribed ADHD medication could be affected by differences in clinical practice, but these differences are not likely to affect individuals with type 1 diabetes differently from individuals in the general population. Despite these limitations, the study has several strengths. The study provides longitudinal data and uses register linkage from national registers with high data completeness. The risk of selection bias due to social inequalities is probably smaller in Norway than in many other countries since the right to health care is universal and paid for by the government. The accuracy of HbA1c measurement, which was analysed at a single DCCT-standardised laboratory, adds to the strength of our study. Finally, by including separate analyses for males and females, we revealed important sex differences.

## 5. Conclusions

First, the prevalence of prescribed ADHD medication was higher, and the age of first prescription was lower in males than in females. One may wonder whether the risk of not recognising an underlying ADHD is higher in females than males with type 1 diabetes and high HbA1c, since females are also more likely to present with the inattentive type of ADHD.

Second, the mean HbA1c in individuals improved during the last decade, but HbA1c continued to be higher in individuals with a comorbid ADHD diagnosis than in individuals with type 1 diabetes only. Individuals with dual diagnosis seem to struggle to achieve good metabolic control, and they are more likely to use insulin pumps. Our findings support the assumption that individuals with a dual diagnosis need special support to manage their diabetes treatment and to fully benefit from advances in diabetes technology [1, 3, 27].

## Figures and Tables

**Table 1:** Annual period prevalence of prescribed ADHD medications, 2005–2019, in the general paediatric population in Norway and in the paediatric population with type 1 diabetes in the Norwegian Childhood Diabetes Registry.

	General Paediatric Population in Norway								Population with Type 1 Diabetes in the Norwegian Childhood Diabetes Registry							
Year	Number of children 5–19 years	Number treated with ADHD medications	Percentage of individuals treated with ADHD medications						Number of children 5–19 years	Number treated with ADHD medications	Percentage of individuals treated with ADHD medications					
			Total	Female	Male	5–9 years	10–14 years	15–19 years			Total	Female	Male	5–9 years	10–14 years	15–19 years
2005	916,026	12,096	1.32	0.61	2.00	0.71	1.86	1.38	133	3	2.26	1.52	2.99	—	4.47	—
2006	922,496	13,181	1.43	0.71	2.11	0.68	1.99	1.60	342	3	0.88	1.14	0.60	—	1.16	2.22
2007	926,476	14,609	1.58	0.82	2.29	0.72	2.17	1.79	570	7	1.23	0.72	1.71	1.09	1.37	1.05
2008	929,785	15,830	1.70	0.93	2.44	0.74	2.32	1.99	853	12	1.52	1.00	1.99	0.39	1.68	2.25
2009	932,392	16,806	1.80	1.01	2.55	0.76	2.49	2.09	1,154	20	1.82	1.10	2.45	1.22	2.17	1.47
2010	933,137	17,516	1.88	1.07	2.64	0.79	2.59	2.19	1,401	32	2.28	1.30	3.05	0.79	2.40	3.56
2011	934,761	17,590	1.88	1.08	2.64	0.74	2.63	2.22	1,646	34	2.07	1.41	2.65	0.73	2.38	2.73
2012	936,840	17,772	1.90	1.11	2.65	0.76	2.67	2.23	1,795	38	2.12	1.52	2.67	1.12	2.02	3.07
2013	941,449	17,817	1.89	1.11	2.64	0.74	2.70	2.23	1,951	38	1.95	1.18	2.66	1.54	1.92	2.27
2014	947,178	18,214	1.92	1.15	2.66	0.77	2.74	2.27	2,158	42	1.95	1.17	2.66	1.61	2.78	1.10
2015	951,811	18,372	1.93	1.18	2.64	0.78	2.75	2.30	1,869	40	2.14	1.34	2.87	1.02	3.33	1.36
2016	955,133	18,692	1.96	1.19	2.69	0.80	2.76	2.35	2 361	56	2.37	1.73	2.93	1.27	3.32	1.92
2017	958,395	19,349	2.02	1.22	2.77	0.85	2.83	2.40	2,497	66	2.64	1.76	3.38	2.06	3.15	2.42
2018	958,780	19,687	2.06	1.27	2.80	0.82	2.86	2.48	2,612	67	2.57	1.91	3.13	1.76	2.74	2.85
2019	956,963	20,299	2.12	1.33	2.88	0.82	2.97	2.55	2,696	69	2.56	1.91	3.13	1.39	3.26	2.50

**Table 2:** Characteristics of the total number of individuals included in the study at their first annual registration (between 2005 and 2022) in the National Childhood Diabetes Registry (NCDR).

Patient characteristics	Type 1 diabetes without comorbid ADHD			Type 1 diabetes with comorbid ADHD			*p*-Value (total)
	Total	Male	Female	Total	Male	Female	
*N*	5888	3195 (54%)	2693 (46%)	365	242 (66%)	123 (34%)	—
Age, years							
Mean (SD)	10.5 (4.1)	10.8 (4.2)	10.2 (4.0)	10.9 (3.9)	11.0 (4.0)	10.7 (3.6)	0.072
Diabetes duration, years							
Mean (SD)	1.2 (0.8)	1.2 (0.8)	1.2 (0.7)	1.1 (0.6)	1.1 (0.6)	1.1 (0.7)	0.330
HbA1c, mmol/mol							
Mean	58.2 (12.8)	57.7 (12.7)	58.9 (12.9)	59.8 (14.3)	59.6 (13.7)	60.4 (15.5)	0.040^a^
Body Mass Index (BMI)							
Mean (SD)	19.0 (3.7)	19.0 (3.7)	18.9 (3.6)	19.3 (3.6)	19.3 (3.5)	19.2 (3.7)	0.606

Abbreviation: SD, standard deviation.

^a^Significant in total and in males, but not females, with/without comorbid ADHD.

## Data Availability

The registry data that has been used for this study has not been made available due to the ethics rules of the Norwegian Childhood Diabetes Registry (NCDR) and for reasons of patient privacy.

## Supporting Information

Supporting Information Table S1: Annual period prevalence of prescribed ADHD medications 2005–2019 with 95% confidence intervals (CIs).
