# Supplementary material for: Comorbidity of Type 1 Diabetes and ADHD: A Longitudinal Cohort Study in Males and Females From the Norwegian Childhood Diabetes Registry
Source: Pediatr Diabetes. 2025 Nov 10;2025:9574797. doi: 10.1155/pedi/9574797 (PMC12623084; doi:10.1155/pedi/9574797)
Supplement: Supporting Information — Table S1: Annual period prevalence of prescribed ADHD medications 2005–2019 with 95% confidence intervals (CIs). [file 9574797.f1.docx]

**Supplementary table 1.** Annual period prevalence of prescribed ADHD medications 2005-2019.

| **Year** | **General paediatric population in Norway** | **Population with type 1 diabetes in the Norwegian Childhood Diabetes Registry** |
| --- | --- | --- |
| 2005 | 1.32 (1.30, 1.34) | 2.26 (0.77, 6.42) |
| 2006 | 1.43 (1.41, 1.45) | 0.88 (0.30, 2.55) |
| 2007 | 1.58 (1.55, 1.60) | 1.23 (0.60, 2.51) |
| 2008 | 1.70 (1.68, 1.73) | 1.52 (0.81, 2.44) |
| 2009 | 1.80 (1.78, 1.83) | 1.82 (1.13, 2.66) |
| 2010 | 1.88 (1.85, 1.93) | 2.28 (1.62, 3.21) |
| 2011 | 1.88 (1.85, 1.91) | 2.07 (1.48, 2.87) |
| 2012 | 1.90 (1.87, 1.93) | 2.12 (1.55, 2.90) |
| 2013 | 1.89 (1.87, 1.92) | 1.95 (1.42, 2.66) |
| 2014 | 1.92 (1.90, 1.95) | 1.95 (1.44, 2.62) |
| 2015 | 1.93 (1.90, 1.96) | 2.14 (1.58, 2.90) |
| 2016 | 1.96 (1.93, 1.99) | 2.37 (1.83, 3.07) |
| 2017 | 2.02 (1.99, 2.05) | 2.64 (2.08, 3.35) |
| 2018 | 2.06 (2.03, 2.08) | 2.57 (2.03, 3.25) |
| 2019 | 2.12 (2.09, 2.15) | 2.56 (2.03, 3.23) |

Numbers are prevalence in percentage with 95% confidence intervals (CIs), and we have used www.openepi.com.
